# Genetic relationships among seven sections of genus *Arachis *studied by using SSR markers

**DOI:** 10.1186/1471-2229-10-15

**Published:** 2010-01-20

**Authors:** Ravi Koppolu, Hari D Upadhyaya, Sangam L Dwivedi, David A Hoisington, Rajeev K Varshney

**Affiliations:** 1 International Crops Research Institute for the Semi-Arid Tropics (ICRISAT), Patancheru, Greater Hyderabad 502 324, AP, India; 2 Genomics towards Gene Discovery Sub Programme, Generation Challenge Programme (GCP), c/o CIMMYT, Int APDO Postal 6-641, 06600 Mexico DF, Mexico

## Abstract

**Background:**

The genus *Arachis*, originated in South America, is divided into nine taxonomical sections comprising of 80 species. Most of the *Arachis *species are diploids (2*n *= 2*x *= 20) and the tetraploid species (2*n *= 2*x *= 40) are found in sections *Arachis*, *Extranervosae *and *Rhizomatosae*. Diploid species have great potential to be used as resistance sources for agronomic traits like pests and diseases, drought related traits and different life cycle spans. Understanding of genetic relationships among wild species and between wild and cultivated species will be useful for enhanced utilization of wild species in improving cultivated germplasm. The present study was undertaken to evaluate genetic relationships among species (96 accessions) belonging to seven sections of *Arachis *by using simple sequence repeat (SSR) markers developed from *Arachis hypogaea *genomic library and gene sequences from related genera of *Arachis*.

**Results:**

The average transferability rate of 101 SSR markers tested to section *Arachis *and six other sections was 81% and 59% respectively. Five markers (IPAHM 164, IPAHM 165, IPAHM 407a, IPAHM 409, and IPAHM 659) showed 100% transferability. Cluster analysis of allelic data from a subset of 32 SSR markers on 85 wild and 11 cultivated accessions grouped accessions according to their genome composition, sections and species to which they belong. A total of 109 species specific alleles were detected in different wild species, *Arachis pusilla *exhibited largest number of species specific alleles (15). Based on genetic distance analysis, the A-genome accession ICG 8200 (*A. duranensis*) and the B-genome accession ICG 8206 (*A. ipaënsis*) were found most closely related to *A. hypogaea*.

**Conclusion:**

A set of cross species and cross section transferable SSR markers has been identified that will be useful for genetic studies of wild species of *Arachis*, including comparative genome mapping, germplasm analysis, population genetic structure and phylogenetic inferences among species. The present study provides strong support based on both genomic and genic markers, probably for the first time, on relationships of *A. monticola *and *A. hypogaea *as well as on the most probable donor of A and B-genomes of cultivated groundnut.

## Background

The genus *Arachis *has its origin in South America where the species of this genus are widespread [1]. This genus includes 80 species, 69 species described by Krapovickas and Gregory [1] and 11 species described by Valls and Simpson [2]. *Arachis *is divided into 9 sections (*Arachis*, *Erectoides*, *Heteranthae*, *Caulorrhizae*, *Rhizomatosae*, *Extranervosae*, *Triseminatae*, *Procumbentes *and *Trierectoides*) based on morphology, geographic distribution and cross compatibility relationships [1]. Species present in sections *Erectoides*, *Extranervosae *and *Triseminatae *and diploid species of section *Rhizomatosae *are believed to be basal in their divergence when compared to the species in other sections [3,4]. Section *Arachis *has 31 species out of the 80 species described. *Arachis *has species cultivated for seeds and pods *Arachis hypogaea*, forage species *Arachis pintoi*, *Arachis glabrata *and *A. sylvestris *[5] and ornamental species *Arachis repens *[6].

Nearly all *Arachis *species are diploid (2*n *= 2*x *= 20), but the cultivated groundnut is an allotetraploid (AABB) (2*n *= 4*x *= 40) and is a member of the section *Arachis*, which also includes another allotetraploid wild species, *Arachis monticola*, which is the probable wild ancestor of *Arachis hypogaea *[1,2]. Apart from section *Arachis*, tetraploid species are also found in sections *Extranervosae *and *Rhizomatosae *(*A. glabrata*, *A. pseudovillosa *and *A. nitida*). Tetraploids in section *Rhizomatosae *appear to have similarities to the genomes of species in sections *Erectoides *and *Arachis *[7]. Along with diploid and tetraploid species, three aneuploid species (2*n *= 2*x *= 18) (*A. decora*, *A. palustris *and *A. praecox*) are also present in this genus [8,9]. Diploid species have received particular attention because they have great potential to be used as sources for several agronomic traits, including resistance to a variety of pests and diseases, drought resistance and different life-cycle spans [6,10-12].

The cultivated species has arisen probably from a unique cross between the wild diploid species *Arachis duranensis *(A-genome) and *Arachis ipaënsis *(B-genome) resulting in a hybrid whose chromosomal number was spontaneously duplicated [13]. The hybridization and chromosome duplication isolated cultivated groundnut from its wild diploid relatives and natural introgression of alleles from wild species into cultivated species has not been demonstrated [14]. Thus the origin through a single and recent polyploidization event, followed by successive selection resulted in a highly conserved genome [15]. Polyploidy in sections *Arachis*, *Extranervosae *and *Rhizomatosae *is believed to have originated independently [16]. Apart from A-genome and B-genome species section *Arachis *has a lone D-genome species *A. glandulifera *[17].

Even though extensive levels of morphological variations are observed in *Arachis hypogaea*, which is most probably due to the variation in few genes [18], molecular markers have shown little polymorphism in the germplasm of this species [18-20]. The low level of polymorphism observed in *A. hypogaea *can be attributed to: (i) barriers to the gene flow from related diploid species to domesticated groundnut as a consequence of the polyploidization event [15], (ii) recent polyploidization from one or a few individuals of each diploid parental species, combined with self pollination [21], (iii) narrow genetic base of cultivated germplasm due to use of few elite breeding lines and little exotic germplasm in breeding programs [22,23] and (iv) unavailability of suitable molecular marker system [24].

Microsatellites or simple sequence repeats (SSRs) have become one of the important classes of molecular markers in the recent past due to their high information content and other features such as high reproducibility and co-dominance [25]. As a result of considerable efforts of several research groups at an international level, several hundred SSR markers have become available in groundnut (see review by Varshney and colleagues) [24]. These SSR markers are very useful to detect genetic variability in groundnut germplasm including cultivated and wild genotypes [26-29] and have been used in the preparation of genetic linkage maps for diploid [30,31] and tetraploid genomes [32] of groundnut. The majority of the SSR markers developed to date are derived from the cultivated tetraploid (AB-genome) groundnut using genomic DNA libraries. As development of SSR markers from genomic DNA libraries is a labor intensive and expensive task, EST (Expressed Sequence Tags) or gene sequences from the species or even related species have also been used to develop SSR markers in groundnut [27,33]. SSR markers derived from genomic DNA or gene sequences have been found useful to assess genetic variability in wild *Arachis *germplasm, which exhibit more variability compared to cultivated species [26,33,34].

The present study was undertaken to determine genetic relationships among 96 accessions of 36 different species and 7 sections of *Arachis *by using the SSR markers developed by Cuc and colleagues [28] from genomic DNA library of cultivated groundnut and SSR markers developed from gene sequences of aeschynomenoid/dalbergoid and genistoid clades of leguminosae family by Mace and colleagues [27].

## Results

### Cross species transferability of SSR primer pairs

In the present study 82 *Arachis hypogaea *SSRs (*Ah *SSRs) developed by Cuc and colleagues [28] and 19 cross species SSRs (*CS *SSRs) developed by Mace and colleagues [27] were tested on 85 accessions of 35 wild *Arachis *species and 11 cultivated *A. hypogaea *accessions, representing 7 different sections of the genus *Arachis*. Cross species transferability was scored positively only when sharp band(s) were present. The average transferability rate of 101 primer pairs (*Ah *SSRs and *C*S SSRs) tested was 81% to section *Arachis*, but ranged from 44% (*Triseminatae*) to 73% (*Erectoides*), to other six sections with an average of 59% (See Additional file 1).

On an average, *CS *SSRs showed higher transferability to accessions of different sections analyzed (88%) compared to *Ah *SSRs (76%) (Table 1). Five primer pairs namely IPAHM 164, IPAHM 165, IPAHM 407a, IPAHM 409 and IPAHM 659 were transferable to all the accessions of different species tested. Some primer pairs showed an interesting feature of amplification in most of the accessions but no amplification in accessions of one section or species. For example the primer pair IPAHM 466 did not show amplification in any of the 17 accessions studied in section *Heteranthae*, while it showed amplification in accessions of all other sections studied; this suggests either the mutation or small deletion in primer binding sites of the primer pair for this SSR locus or complete absence of this SSR locus in section *Heteranthae*. On the other hand, IPAHM 176 was not transferable to sections *Caulorrhizae*, *Triseminatae *and *Extranervosae *while it was transferable to all the other sections. Apart from A, B, AB and D-genome species of section *Arachis *which are phylogenetically closely related, 88 primer pairs were transferable to species of section *Erectoides *and 54 primer pairs were transferable to species of section *Extranervosae *(See Additional file 1). The number of primer pairs transferable to species belonging to other sections is given in the Additional file 1. Sizes of the fragments amplified by the primer pairs were usually similar to those of the donor species, suggesting that the amplicons were derived from the same loci and that these allelic regions of the primer binding sites are conserved, but 7 primer pairs (37%) tested out of 19 *CS *SSR primer pairs, amplified products of higher molecular weight in some of the accessions tested.

**Table 1:** Transferability rates of *Ah *SSRs and *CS *SSRs to different sections of *Arachis*

S.N.	Section	Number of transferable *Ah *SSRs (%)	Number of transferable *CS *SSRs (%)
1	*Arachis*	82 (100)	19 (100)
2	*Caulorrhizae*	56 (68)	17 (89)
3	*Erectoides*	71 (87)	18 (95)
4	*Heteranthae*	69 (84)	19 (100)
5	*Procumbentes*	69 (84)	19 (100)
6	*Triseminatae*	49 (60)	12 (63)
7	*Extranervosae*	41 (50)	13 (68)
	Mean	62 (76)	17 (88)

S.N.: Serial Number

*Ah *SSRs: *Arachis hypogaea *SSRs

*CS *SSRs: Cross Species SSRs

### Alleles specific to different *Arachis *species

Based on the amplification events in *A. duranensis *(A-genome), *A. ipaënsis *(B-genome) and *A. hypogaea *(AB-genome) the primer pairs tested in the present study can be grouped into three classes: (a) primer pairs amplifying *A. duranensis *and *A. ipaënsis *and amplifying putative alleles of both species in *A. hypogaea*; (b) primer pairs amplifying only *A. duranensis *but not *A. ipaënsis *and amplifying putative *A. duranensis *allele in *A. hypogaea*; (c) primer pairs amplifying *A. ipaënsis *but not *A. duranensis *and amplifying putative *A. ipaënsis *allele in *A. hypogaea*. Majority of the primer pairs tested fell into the first class mentioned above. Since there has been a controversy over the probable B-genome donor for cultivated groundnut, our results strengthen the hypothesis that *A. ipaënsis *is the most probable B-genome donor for the cultivated groundnut. Some primer pairs amplified alleles specific to a particular species. For example in case of *CS *SSRs, the primer pair 68_Stylo_SSR1-24, amplified a unique 600 bp allele in species belonging to *A. batizocoi *(B-genome, section *Arachis*) which is not present in any other species tested with this primer pair, the same primer pair has amplified a unique 580 bp allele in *A. pintoi *(section *Caulorrhizae*) which is not present in any other species tested. The list of primer pairs (39) amplifying a number of unique alleles in different species and corresponding sections are given in the Additional file 2. *A. pusilla *exhibited largest proportion of species specific alleles (15, 14%, section *Heteranthae*). Most of the species studied using the SSR primer pairs exhibited unique alleles, indicating the wide genetic base of the *Arachis *species. *A. diogoi*, *A. kempff-mercadoi *and *A. ipaënsis *belonging to section *Arachis*, *A. hermannii *belonging to section *Erectoides *and *A. kretschmeri *and *A. subcoriacea *belonging to section *Procumbentes *did not show species specific alleles.

### Alleles specific to different *Arachis *sections

Species belonging to section *Arachis *showed the largest number of specific alleles (48, 47%), followed by section *Heteranthae *(29, 29%) and species belonging to sections *Triseminatae *and *Extranervosae *showed least number of specific alleles (3, 3%). Four primer pairs IPAHM 461, IPAHM 455, IPAHM 373 and IPAHM 334 amplified fragments only in species of section *Arachis *with A-genome, B-genome, AB-genome and or D-genome indicating that they are specific to section *Arachis*. The primer pair IPAHM 451, which is derived from genomic DNA library of cultivated groundnut amplified in AB-genome species but alleles corresponding to the A-genome and B-genome were not observed in wild diploid progenitor species indicating that this microsatellite locus/region was created after the polyploidization event.

### Locus duplication

Ten (IPAHM 531, IPAHM 91, IPAHM 105, IPAHM 117, IPAHM 176, IPAHM 183, IPAHM 320, IPAHM 377, IPAHM 659 and IPAHM 695) out of the 101 primer pairs tested amplified duplicated loci in tetraploid accessions of *Arachis *section. The primer pair IPAHM 531, amplified 320 and 325 bp fragments in *A. duranensis *(A-genome), in *A. ipaënsis *(B-genome) it amplified a 300 bp fragment and in the tetraploid *A. hypogaea *and *A. monticola *accessions both 320 and 300 bp fragments were amplified, apart from these two fragments, another fragment with 900 bp size was amplified in all the 11 accessions of cultivated tetraploid species *A. hypogaea *and lone accession of wild tetraploid species *A. monticola *(ICG 13177). The 900 bp fragment amplified was unique to tetraploid accessions and was not observed in diploid species. Primer pair IPAHM 117 amplified a 200 bp fragment in both *A. duranensis *and *A. ipaënsis *accessions and all the tetraploid accessions, apart from this two additional fragments with sizes 400 bp (in ICG 7827, ICG 156, ICG 12625, ICG 12719, ICG 9930, ICG 2738, ICG 13942, ICG 15207, ICG 15206, ICG 10044, ICG 7893, ICG 13177) and 405 bp (in ICG 9930, ICG 2738) were amplified in tetraploid accessions, which were not observed in diploid species. The remaining primer pairs also amplified a unique second locus that was specific to tetraploid accessions. All the primer pairs that amplified a duplicated locus in tetraploid accessions in this study belonged to *Ah *SSRs developed by Cuc and colleagues [28] and none to *CS *SSRs developed by Mace and colleagues [27].

### Genetic diversity and relationships among the wild *Arachis *germplasm

**Figure 1:**
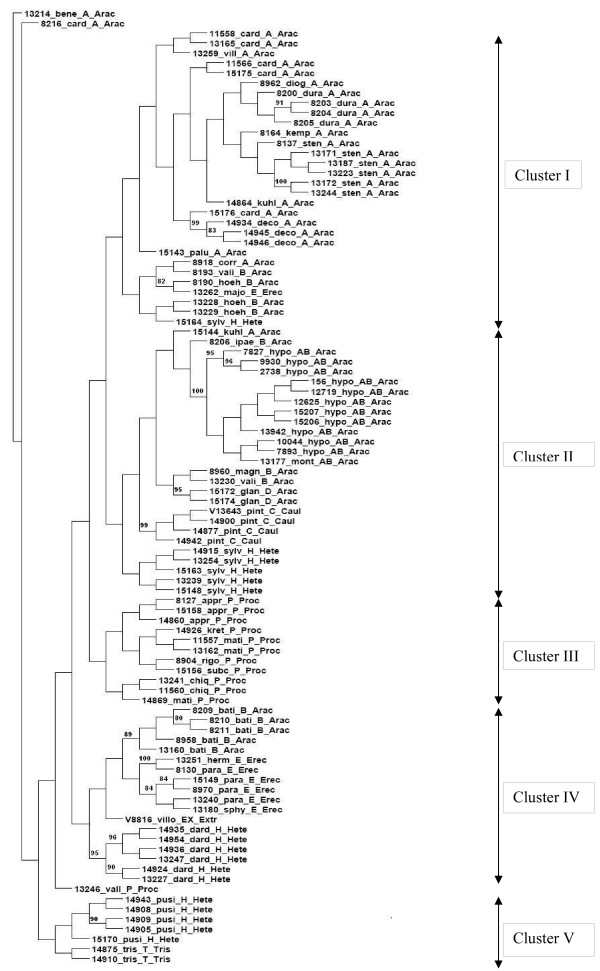
**Dendrogram of wild and cultivated *Arachis *accessions based on SSR polymorphism**. Cluster analysis was performed using the neighbor-joining method. Bootstrap values obtained from 1000 replicate analyses higher than 80% are indicated on nodes. The names of accessions and taxonomical information are given next to their branches starting with the accession number followed by an abbreviated form of species name followed by respective genomes and sections (Abbreviated species names: bene: *benensis; *card: *cardenasii*; diog: *diogoi*; dura: *duranensis*; kemp: *kempff-mercadoi*; sten: *stenosperma*; kuhl: *kuhlmannii*; deco: *decora*; palu: *palustris*; corr: *correntina*; vali: *valida*; hoeh: *hoehnei*; majo: *major*; sylv: *sylvestris*; ipae: *ipaënsis*; hypo: *hypogaea*; mont: *monticola*; magn: *magna*; glan: *glandulifera*; pint: *pintoi*; appr: *appressipila*; kret: *kretschmeri*; mati: *matiensis*; rigo: *rigonii*; subc: *subcoriacea*; chiq: *chiquitana*; mati: *matiensis*; bati: *batizocoi*; herm: *hermannii*; para: *paraguariensis*; sphy: *stenophylla*; vill: *villosa*; pusi: *pusilla*; dard: *dardani*; vall: *vallsii*; tris: *triseminata*). (Abbreviated section names: Arac: *Arachis*; Caul: *Caulorrhizae*; Hete: *Heteranthae*; Proc: *Procumbentes*; Erec: *Erectoides*; Tris: *Triseminatae*; Extr: *Extranervosae*)

The *Arachis *germplasm surveyed by SSR analysis consisted of 96 accessions representing 36 different species from sections *Arachis*, *Caulorrhizae*, *Heteranthae*, *Procumbentes*, *Erectoides*, *Triseminatae *and *Extranervosae*. This set included 11 accessions of *A. hypogaea *that helped in comparative analysis of wild accessions with cultivated accessions. Although the level of transferability observed varied among the101 primer pairs used, only 32 primer pairs showed amplification in at least 70% of accessions analyzed in the study. Therefore, genotyping data for 454 alleles obtained with these 32 primer pairs (Table 2) were used for constructing dendrogram by using NJ-method (Figure 1). The dendrogram showed that all but seven wild *Arachis *accessions were grouped mainly according to their genomes and sections. The seven accessions that were not grouped by sections were: ICG 13262 (*A. major*, section *Erectoides*), ICG 15164 (*A. sylvestris*, section *Heteranthae*), ICG 8209, ICG 8210, ICG 8211, ICG 8958 and ICG 13160 (*A. batizocoi*, section *Arachis*). Out of the 36 species studied, except a few accessions of *A. valida *(ICG 8193, ICG 13230), *A. kuhlmannii *(ICG 14864, ICG 15144), *A. sylvestris *(ICG 15164) and *A. cardenasii *(ICG 15176), all the other accessions were grouped with respect to their species. For the accessions belonging to these species, intraspecific variation was high, and as a result, not all the accessions have clustered together.

**Table 2:** List of SSR markers used for cluster analysis

S.N.	Marker	Number of Alleles
1	04_Dal_PHYA	9
2	09_Lup_CycB	29
3	12_Lup_ACS2	28
4	34_Lup_app	23
5	63_Stylo_IGS	8
6	66_Stylo_SSR4-5	7
7	69_Stylo_IGS	4
8	68_Stylo_SSR1-24	10
9	76_Stylo_IGS	7
10	IPAHM117	11
11	IPAHM130	8
12	IPAHM164	15
13	IPAHM165	16
14	IPAHM273	10
15	IPAHM320	17
16	IPAHM372	18
17	IPAHM357	15
18	IPAHM377	13
19	IPAHM407a	17
20	IPAHM409	11
21	IPAHM171c	16
22	IPAHM109	8
23	IPAHM324	8
24	IPAHM105	18
25	IPAHM414	15
26	IPAHM288	20
27	IPAHM82	20
28	IPAHM606	16
29	IPAHM176	16
30	IPAHM395	13
31	IAPHM245	8
32	IAPHM406	20

The dendrogram has grouped different taxa into five main clusters. Cluster I contained accessions of all A-genome species (*A. cardenasii*, *A. villosa*, *A. diogoi*, *A. duranensis*, *A. kempff-mercadoi*, *A. stenosperma*, *A. kuhlmannii *and *A. correntina*), accessions of two B-genome species (*A. valida *and *A. hoehnei*), one accession each of sections *Erectoides *(*A. major*) and *Heteranthae *(*A. sylvestris*) together with ICG 13262 and ICG 15164 discussed above. Cluster II contained accessions of all tetraploid AB-genome species, one accession of A-genome species (*A. kuhlmannii*), one accession each of three B-genome species (*A. ipaënsis*, *A. magna *and *A.valida*), all the accessions of D-genome species (*A. glandulifera*), all four accessions of *A. pintoi *(section *Caulorrhizae*) and five accessions of *A. sylvestris *(section *Heteranthae*). Cluster III included exclusively the species of section *Procumbentes*, *A. kretschmeri*, *A. matiensis*, *A. rigonii*, *A. subcoriacea *and *A. chiquitana*. Cluster IV consisted of all the five accessions of B-genome species (*A. batizocoi*), three species of section *Erectoides *(*A. hermannii*, *A. paraguariensis *and *A. stenophylla*), the lone included species of section *Extranervosae *(*A. villosulicarpa*) and all the six accessions of *A. dardani *(section *Heteranthae*) together with the 5 misplaced *Arachis batizocoi *accessions. Cluster V was formed by all the accessions of *A. pusilla *(section *Heteranthae*) and *A. triseminata *(section *Triseminatae*). Three accessions ICG 13214 (*A. benensis*), ICG 8216 (*A. cardenasii*) and ICG 13246 (*A. vallsii*), however, could not be grouped in any cluster/sub-cluster.

**Figure 2:**
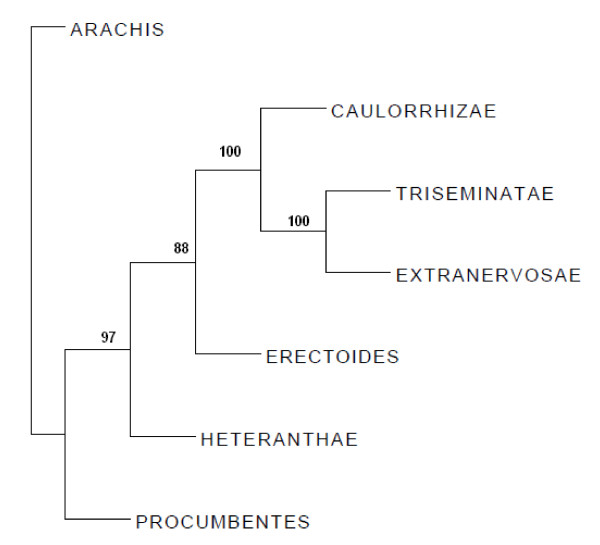
**Dendrogram of seven sections of *Arachis***. Allelic data based on 32 SSR markers was used to develop dendrogram. The numbers on the nodes indicate bootstrap values for grouping based on 1000 bootstrap replicates.

The dendrogram constructed for different sections based on allelic data from 32 SSRs could differentiate the seven sections of *Arachis *(Figure 2). Sections *Erectoides*, *Triseminatae *and *Extranervosae*, which are believed to be basal in their divergence, were clustered close to each other and the sections *Arachis *and *Procumbentes *have diverged into two separate out groups.

### Probable genome donors of cultivated groundnut

In order to verify the most probable genome donors for A-genome and B-genome of cultivated groundnut, the genetic distance between each diploid accession and all *Arachis hypogaea *accessions as a group was calculated. The distances obtained were compared to find the accessions more closely related to the tetraploid accessions. Among all the A-genome species accessions tested, accessions ICG 8200 (*A. duranensis*, distance: 0.114), ICG 8962 (*A. diogoi*, distance: 0.115), ICG 8204 (*A. duranensis*, distance: 0.117) have shown least distances to *A. hypogaea*. Among accessions of all B-genome species, accessions ICG 8206 (*A. ipaënsis*, distance: 0.083), ICG 8211 (*A. batizocoi*, distance: 0.104) and ICG 8209 (*A. batizocoi*, distance: 0.112) showed least distances to *A. hypogaea*. Genetic distances between other A-genome and B-genome species, *A. hypogaea* and *A. monticola* are given in Additional file 3. Clustering of the accessions also revealed that the lone accession of *A. ipaënsis* clustered closely with *A. hypogaea* accessions, suggesting *A. ipaënsis* as the most probable B-genome donor.

## Discussion

### SSR transferability

The transferability of SSR information from one species to a related second species can be defined as the probability of success in PCR amplification using heterologous primer pairs designed for the first species [35]. Transferability of SSR markers between related species is a consequence of the homology of flanking sequences of the microsatellites and size of the region between the primer pairs amenable to amplification by PCR. Previous studies have demonstrated the conservation of SSR sequences in plants [25,36]. The donor source of primer pairs influences levels of transferability, which reflect the genetic distance between the donor and target species. Apart from this, some other factors such as mutations in flanking regions, ploidy level [37], large intronic regions in case of genic SSRs, template DNA concentration and PCR conditions used may complicate the relationship of transferability.

The present study employed a total of 82 *Ah *SSR primer pairs and 19 *CS *SSR primer pairs for transferability across seven sections and 36 species of *Arachis*. In general *CS *SSR markers showed higher transferability to accessions of different sections compared to *Ah *SSRs. This difference was found statistically significant based on Z-test (Z= 3.59, *P < 0.05*). This can be attributed to higher conservation in genic regions from where *CS *SSR markers were developed [27] as compared to less conserved genomic regions from where *Ah *SSR markers were developed [28]. Several other studies in past reported the cross transferability of *Arachis hypogaea *SSR markers in different species/sections of *Arachis*. For instance, Hopkins and colleagues [14] observed cross species amplification of *Arachis hypogaea *SSR markers in *Arachis monticola*, *Arachis duranensis *and *Arachis ipaënsis*. Moretzsohn and colleagues [26] observed cross species amplification of *Arachis hypogaea *SSR markers in different sections of *Arachis *(76% to species of section *Arachis *and 45% to species of the other eight sections). Similarly, Gimenes and colleagues [34] also observed a cross transferability rate of 60 to 100% to species belonging to different sections of *Arachis *using *Arachis hypogaea *microsatellites. The higher rate of transferability observed in our study can be attributed to the use of larger number of SSR markers, and also the conserved genic regions as origin of *CS *SSRs used.

Most of the primer pairs tested in the present study amplified regions of expected product size in different *Arachis *species. The reason for this can be explained by the conservation of flanking regions and repeat sequences in majority of the *Arachis *species, but seven out of the twelve *CS *EST-SSR primer pairs amplified products of higher molecular weight. The increase in product size when using EST-SSRs is reasonable, because the amplification is based on genomic DNA, which may contain non-coding intronic regions between exons and get amplified by using the primer pairs designed from EST/genic sequences. Also the PCR fragments amplified by using *CS *SSR markers were of better quality with strong and distinct allelic bands than those obtained by *Ah *SSR markers which have the problems like stuttering and faint bands. Better quality and larger size of products than expected has been a characteristic feature of genic/EST derived SSR markers [36].

The average transferability rate of all the primer pairs tested was higher to species of section *Arachis *presumably because species belonging to section *Arachis *are phylogenetically more closely related to each other and the source of most SSRs studied is section *Arachis*. The least transferability rate was observed in case of section *Triseminatae *a basally diverged species [3,4]. Apart from these less transferability was also observed in section *Heteranthae*. The primer pairs IPAHM 164, IPAHM 165, IPAHM 407a, IPAHM 409 and IPAHM 659 were transferable to all the accessions of different species studied. This suggests that these SSRs arose before speciation and are positioned in or near coding regions with a conserved sequence across species. As these five primer pairs were transferable to accessions of all diploid species these can serve as informative markers in wild *Arachis *species. The primer pairs IPAHM 461, IPAHM 455, IPAHM 373 and IPAHM 334 were transferable only to section *Arachis*, which comprise of A-genome, B-genome, AB-genome and D-genome species.

The use of SSR markers developed for one species in genetic evaluation of other species considerably reduces the time and cost involved in SSR development, since the development of SSRs is expensive and time consuming. The transferable SSR markers identified in our study could be very useful for genetic analysis of wild species of *Arachis*, including comparative genome mapping [35,38], population genetic structure and phylogenetic inferences among different species. For using SSR markers of one species in the evaluation of other species, our study recommends first to screen a large number of SSR markers developed in a particular species and then identify the subset of most reliable markers that amplify the expected amplicons in the other species. The number of cross transferable SSRs can be increased by using SSR markers derived from EST or genic sequences [36].

### *Arachis hypogaea *- locus duplication

Ten *Ah *SSR primer pairs out of 82 analyzed amplified more than one locus in tetraploid accessions of *Arachis *section indicating loci duplication. For these A- genome fragments as well as B-genome fragments were observed but, one additional fragment was also observed in all the tetraploid accessions. The nature of this extra fragment remains to be determined. Amplification of more than one fragment by one primer pair in tetraploid groundnut accessions has been reported in several other studies [14,29,34]. While developing the genetic linkage map for tetraploid groundnut, Varshney and colleagues [32] identified duplicated loci in the segregating population for at least 5 markers and in case of the marker TC3G01 they identified 3 different scorable segregating loci in the population. Amplification of more than one fragment by a primer pair can also be due to heterozygosity. However *Arachis hypogaea *is an allotetraploid, and preferentially an autogamous species with a cross-pollination rate of 2.5% [39].

### Alleles specific to species and sections

Out of 101 primer pairs tested 39 primer pairs amplified 109 alleles, which are specific to different species and sections of *Arachis*. For example *A. pusilla *exhibited the largest proportion of species specific alleles (15, 14%, section *Heteranthae*). It is also interesting to note that accessions of most of the species that have amplified unique alleles were originated from Brazil. According to Stalker and colleagues [40], the centre of genetic variation for the genus *Arachis *is the Mato Grosso region of Brazil to eastern Bolivia. However, when specifically comparing *A. hypogaea *to other species, the greatest probability of finding unique genes is in the North-Central, North-East, South and South-East regions of Brazil. Accessions with unique alleles may be useful for introgressing diversity into cultivated groundnut, which has narrow genetic base, for crop improvement. Further evaluation of these novel alleles may provide some association with useful traits for groundnut breeders.

The primer pairs IPAHM 461, IPAHM 455, IPAHM 373 and IPAHM 334 appear to generate SSR alleles specific to species of section *Arachis *which contains A-genome, B-genome, AB-genome and D-genome species. The A-genome is characterized by a small chromosome pair, the "A chromosome" [41]. The A-genome species also have heterochromatic bands in all, or almost all, of their chromosomes and are homogeneous in their gross karyotype structure [42]. The B-genome species have a symmetric karyotype, do not have the "A chromosome" pair. These taxa are more diverse in karyotype formula and in the presence and distribution of heterochromatin [42]. Tetraploid species have an AABB genome constitution, and it has been demonstrated that they originated by hybridization of two wild diploid species, one with the A-genome and the other with the B-genome [43]. The D-genome is another described genome type for the section *Arachis *and it has been proposed to be exclusive to *Arachis glandulifera *[17]. Moretzsohn and colleagues [26] identified one primer pair amplifying alleles specific to A-genome species. These genome specific markers would be useful for identifying DNA fragments introgressed into another species. The primer pair IPAHM 451 has amplified accessions of tetraploid AB-genome species and did not show amplification in their wild progenitors and other species studied. This indicates that these SSRs (genomic regions) probably arose after the polyploidization event, which resulted in tetraploid groundnut. Rapid genomic modifications commonly occur in early generations of newly formed polyploids and harmonize the different genomes in the same nucleus [44]. These may result in either loss or creation of new genomic regions.

### Relationships among the wild *Arachis *germplasm

Knowledge of genetic relationships among various wild species is necessary for successful and efficient exploitation of genetic diversity present in wild species, as the wild species are known to harbor genes for resistance to biotic and abiotic stresses [45,11]. It is easier to transfer these specific genes from the species that are closely related to the focal species than those that are distantly related. In the present study genetic relationships among 96 accessions representing 36 different species from sections *Arachis*, *Caulorrhizae*, *Heteranthae*, *Procumbentes*, *Erectoides*, *Triseminatae *and *Extranervosae *were established using data from 32 SSR markers. In general, the SSR data grouped wild *Arachis *accessions into similar genome/section/species groups with a few exceptions. For example all the accessions of A-, AB- and D-genomes of section *Arachis *and accessions of sections *Caulorrhizae*, *Procumbentes *and *Triseminatae *were clustered. For the species *A. valida*, *A. kuhlmannii*, *A. sylvestris *and *A. hoehnei*, intraspecific variation was high and as a result all the accessions of these species could not be grouped together. The accession ICG 13177 from *A. monticola *species was grouped along with *A. hypogaea *accessions and this grouping was supported by a bootstrap value of 100% (Figure 1). These observations suggest high genetic similarity between *A. monticola *and *A. hypogaea *species and are in agreement with previous studies [46,47,26,34]. Except for IPAHM 93 and IPAHM 373, all the remaining primer pairs tested amplified similar alleles in *A. monticola *and *A. hypogaea *accessions. Earlier studies, by Krapovickas and Gregory [1] reported fertile hybrids from crossing accessions of these two species. Therefore, our study together with earlier studies [48,26] clearly suggests that *A. monticola *could be directly related to the allotetraploid ancestral progenitor of *A. hypogaea*.

The present results agreed with the close relationship between *A. glandulifera*, the lone D-genome species [17] and B-genome species. Tallury and colleagues [49] while studying the sequence data of plastid *trn*T-F region in B-genome and D-genome species identified structural changes in that region that are synamomorphic to the B and D-genomes. They identified a 6-bp indel in this region that is common to both the B and D-genomes, whereas A-genome species have a characteristic 21-bp indel in this region. Gimenes and colleagues [34] also while studying SSR markers in *Arachis *observed close relationship between *A. glandulifera *and B-genome species.

The accessions of *A. decora *(ICG 14934, ICG 14945 and ICG 14946) and *A. palustris* (ICG 15143) both having an aneuploid chromosomal number of 2n = 2x = 18 were grouped close to each other in the present study. Similar results were reported by Bravo and colleagues [47] while studying genetic relationships among different *Arachis* species. These two species are phylogenetically closely related and found to show no polymorphism on their rDNA transcribed spacers [47]. The lone accession of *A. villosulicarpa *ICG 8816 (section *Extranervosae*) was grouped along with species of sections *Erectoides *and *Heteranthae*. These observations indicate the close relationships of species belonging to *Extranervosae *with species of sections *Erectoides *and *Heteranthae*. In the present study some species belonging to different sections were clustered together. For instance, some species belonging to section *Arachis *were clustered with species of section *Erectoides *and some species belonging to section *Erectoides *were grouped along with species of section *Arachis*. Such grouping of species belonging to one section with species belonging to a different section may be attributed to: (i) high levels of polymorphism detected at the analyzed loci, (ii) occurrence of homoplastic alleles, i.e. alleles that present the same size (bp) in a gel, are not identical by descent but identical in their state [36] and are found in relatively distantly related species, and (iii) allele sharing between species belonging to different genomes, and (iv) similar geographic distribution of the accessions belonging to different species analyzed.

Information on genetic relationships among the wild germplasm has implications for potential use of related species in groundnut improvement and also estimates on genetic relatedness can be useful for germplasm conservation efforts, selection of diverse parents for hybridization, and maximizing the range of genetic variability employed in breeding programs.

### Revisiting of genome donors to cultivated groundnut

The cultivated groundnut, *Arachis hypogaea* is believed to be an amphidiploid produced by hybridization of an A-genome species and a B-genome species followed by subsequent chromosomal doubling. Although *A. ipaënsis* was previously proposed as the probable B-genome donor and *A. duranensis* as the probable A-genome donor to the tetraploid *Arachis* species [13,43,50], these propositions have been debatable. Therefore, the obtained results on the accessions of *A. ipaënsis *species in the present study have been critically analyzed.

**Figure 3:**
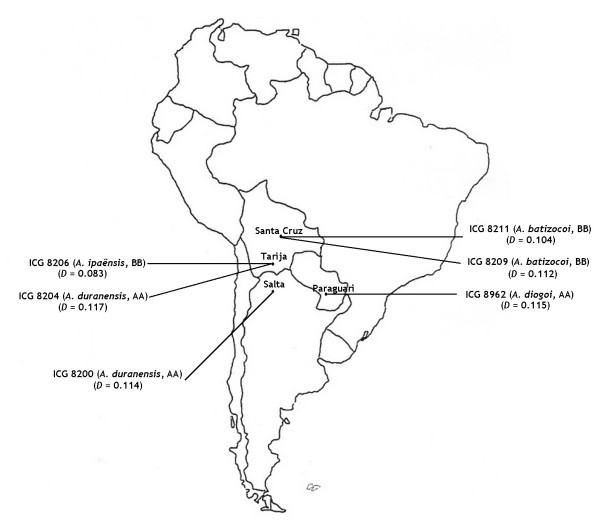
**Approximate geographical locations of A and B-genome accessions**. A few AA-genome and BB-genome species accessions showing the least genetic distance to *Arachis hypogaea* accessions and originated from South America have been shown in the figure. Name of species, genome designation and genetic distance (D) of respective accessions with *A. hypogaea* have been shown in parentheses.

The accession ICG 8206 representing *A. ipaënsis *species was grouped closer to *A. hypogaea *accessions than to the other B-genome species accessions. By using AFLP markers, Tallury and colleagues [49] also found clustering of *A. ipaënsis *accession with *A. hypogaea*. Similarly, based on AFLP and RFLP data, Gimenes and colleagues [51] also demonstrated a close affinity of *A. hypogaea/A. monticola *to *A. ipaënsis *than to *A. duranensis*. Furthermore, among all the B-genome accessions in the present study, the accessions ICG 8206 (*A. ipaënsis*, distance: 0.083), ICG 8211 and ICG 8209 (*A. batizocoi*, distance: 0.104 and 0.112) showed least distances to *A. hypogaea*. These observations indicate a close relationship between them. Similarly, among all the A-genome accessions tested, the accessions ICG 8200 (*A. duranensis*, distance: 0.114), ICG 8962 (*A. diogoi*, distance: 0.115) and ICG 8204 (*A. duranensis*, distance: 0.117) showed least distances to *A. hypogaea*. Therefore, our results strongly support that *A. ipaënsis* and *A. duranensis* are the probable B-genome and A-genome donors. It is also important to note here that *A. hypogaea* is believed to originate from southern Bolivia to northern Argentina. Although the accessions of *A. diogoi* and *A. batizocoi *showed least genetic distances to *A. hypogaea* after *A. duranensis* and *A. ipaënsis* in our study, the geographic location of these accessions does not support their involvement in evolution of *A. hypogaea* (Figure 3). In addition to that, the *A. duranensis* accession ICG 8200, which showed least distance to *A. hypogaea*, is originated from the Salta province of Argentina, the region which is believed to contain A-genome accessions that are most similar to A-genome of *A. hypogaea*[13].

## Conclusions

The present study provides a set of cross species and cross section transferable SSR markers for genetic studies of wild species of *Arachis*, including comparative genome mapping, germplasm analysis, population genetic structure and phylogenetic inferences among species, avoiding the time and cost involved in development of new set of SSR markers. A large number of species/section-specific alleles as well as accessions harboring unique alleles have been identified. This information will be very useful for groundnut community to enhance the genetic base of cultivated groundnut after systematic introgression of diversity from wild species. Results obtained in the present study provided the strong support based on both genomic and genic markers, probably for the first time, on relationships of *A. monticola *and *A. hypogaea *species as well as on the most probable donor of A-genome (*A. duranensis*) and B-genome (*A. ipaënsis*) of cultivated groundnut based on their genetic distances to *A. hypogaea*.

## Methods

### Plant material and DNA extraction

A total of 96 groundnut accessions, which represent 36 species, and 7 sections of the genus *Arachis *were selected for evaluation of genetic relationships and assessing the transferability of SSR markers. Of the 96 accessions 11 accessions represent different botanical types (see Additional file 4) of cultivated groundnut *Arachis hypogaea*, and the remaining 85 accessions represent 35 different species of the genus *Arachis*. These accessions were obtained from RS Paroda Genebank at ICRISAT, Patancheru, India.

Total genomic DNA was isolated from unopened leaves according to a modified CTAB-based procedure [28]. The DNA quality and quantity were checked on 0.8% agarose gels and DNA concentration was normalized to ~5 ng/μl for PCR.

### SSR markers

A total of 101 SSR primer pairs, 82 developed by Cuc and colleagues [28] from the genomic library of tetraploid *Arachis hypogaea *(*Ah *SSRs) and 19 cross species SSR primer pairs (*CS *SSRs) developed by Mace and colleagues [27] by *in silico *mining of gene sequences from aeschynomenoid/dalbergoid and genistoid clades of leguminosae family (see Additional file 5) were selected for assessing the transferability across 7 sections of the *Arachis *genus.

### Polymerase Chain Reaction (PCR)

PCR reactions for all the primer pairs were performed in 5 μl following a touchdown PCR profile in an ABI 9700 thermal cycler (Applied Biosystems, USA). The PCR reaction was performed on ~5 ng of genomic DNA with 2 picomoles of each primer, 2 mM of each dNTP, 2 mM MgCl_2_, 1× amplification buffer (Bioline, USA) and 0.1 U of *Taq *DNA polymerase (Bioline, USA). The touchdown PCR amplification profile has initial denaturation step for 3 min at 94°C followed first by 5 cycles of 94°C for 20 sec, 65°C for 20 sec and 72°C for 30 sec, with 1°C decrease in temperature per each cycle, then followed by 35 cycles of 94°C for 20 sec with constant annealing temperature (59°C) for 20 sec and 72°C for 30 sec, followed by a final extension for 20 min at 72°C. The amplified products were tested on 1.2% agarose gels to check the amplification.

### Electrophoresis and data collection

The PCR products amplified using DNA of wild and cultivated species were electrophoresed on 6% non-denaturing polyacrylamide gels (29:1 acrylamide/bisacrylamide) for 2 h at 800 V and visualized by silver staining.

For checking transferability, data was collected as presence or absence of band at the locus amplified by the particular primer pair. Presence of band was scored as (+) and absence of band was scored as (-). For assessing the genetic relationships among the accessions the amplified products were scored for the presence or absence of alleles. The presence of allele was converted to 1 and the absence of allele to 0. The approximate size of the product was determined based on a 100 bp ladder.

### Analysis of cross species transferability and genetic variation

The transferability of primer pairs was tested using 96 accessions representing 36 species from 7 sections of *Arachis*. Since the *Ah *SSRs were derived from *Arachis hypogaea *genome, accessions of *Arachis hypogaea *used in this study were excluded for the assessment of transferability using these primer pairs, whereas for *CS *SSRs developed by Mace and colleagues [27], *Arachis hypogaea *accessions were included for calculating cross transferability. Percentage transferability was recorded as percentage of amplification of the SSR markers amplified in different accessions tested.

For estimates of genetic diversity among cultivated and wild groundnut germplasm, 11 accessions of cultivated groundnut and 85 accessions of 35 wild species from 7 sections of *Arachis *genus were analyzed. A total of 32 markers were used in this analysis (Table 2). The 0/1 binary matrix of the markers was used for the calculation of genetic distances using Nei and Li [52] distance coefficient and further a Neighbor Joining (NJ) dendrogram was constructed using PAUP* 4.0b10 [53] and Dendroscope [54]. The robustness of the phylogenetic tree was evaluated by bootstrap analysis [55] with 1000 replicates using PAUP* 4.0b10.

## Authors' contributions

RK was involved in generation and analysis of SSR marker data. HDU and SLD selected and provided germplasm analyzed in the study. RKV in consultation with HDU and DAH conceptualized the study, designed experiments and coordinated the study. RK and RKV participated in drafting the manuscript and RKV finalized the manuscript. All authors read and approved the final manuscript.

## Supplementary Material

Additional file 1 **Transferability of the primer pairs tested to species belonging to different sections of *Arachis***. The data provides percentage transferability of each SSR primer pair to different sections of *Arachis*. Click here for file

Additional file 2 **Species and section specific alleles amplified by microsatellites**. The data provides the number of specific alleles amplified by different primer pairs in each species and different sections of *Arachis *studied. Click here for file

Additional file 3 **Genetic distances between the tetraploid *Arachis *species and their diploid wild relatives**. The data provides information on genetic distances between all A, B-genome species studied and AB-genome species (*A. hypogaea *and *A. monticola*). Click here for file

Additional file 4 **List of 96 *Arachis *accessions representing 36 species and 7 sections analyzed**. The data provides information on the accessions used in SSR analysis, their ploidy level, genome composition, species and sections to which they belong to and their geographic origin. Click here for file

Additional file 5 **List of 101 SSR markers used to test transferability rates**. The data provides information about SSR primers used in the study like their source, repeat type, sequence information, and amplification product size. Click here for file
